# Huayu Wan Prevents Lewis Lung Cancer Metastasis in Mice via the Platelet Pathway

**DOI:** 10.1155/2020/1306207

**Published:** 2020-07-27

**Authors:** Yunfei Ma, Guangda Li, Xu Sun, Kexin Cao, Xiaomin Wang, Guowang Yang, Mingwei Yu

**Affiliations:** ^1^Beijing Hospital of Traditional Chinese Medicine, Capital Medical University, Beijing 100010, China; ^2^Beijing University of Chinese Medicine, Beijing 100029, China; ^3^The Tumor Hospital Affiliated to Zhengzhou University, Zhengzhou 450008, China

## Abstract

**Objective:**

To study the mechanism of Huayu Wan on the metastasis of Lewis lung cancer in mice via the platelet pathway.

**Method:**

Construction of the lung metastasis model by injection of Lewis cells through the tail vein. The next day, 72 mice were randomly divided into the Huayu Wan group (HYW), the aspirin group, the control group, and the normal group . Treatment was given for 5 days per week for a total of 16 days. The size and distribution of lung metastases were observed. Thromboelastography was used to detect platelet function, flow cytometry was used to analyze platelet activation, and ELISA was used to detect platelet tumor metastasis-related factor expression.

**Result:**

Lung weight in the control group was significantly higher than that in the HYW group (*P* < 0.05). The distribution of lung metastases in the control group was obviously more than that in the HYW group. The thromboelastogram showed that the *R* value of the control group was significantly lower than the normal group, while the *R* values of the HYW and aspirin groups were higher than the control group (*P* < 0.05). Flow cytometry analysis showed that the expression of CD62P in platelet-rich plasma in the control group was significantly higher than that in the normal group, while the expression of CD62P in the HYW and aspirin groups was lower than that in the control group (*P* < 0.05). In addition, ELISA showed that the expression of VEGF, bFGF, and CD62P in serum of the HYW group was significantly decreased than the control group (*P* < 0.05), and the expression of VEGF and bFGF in serum of the aspirin group was significantly decreased than the control group (*P* < 0.05).

**Conclusion:**

The mechanism of Huayu Wan inhibiting the metastasis of lung cancer in mice may be related to the improvement of blood hypercoagulability, the inhibition of platelet activation, and the expression of VEGF, bFGF, and CD62P.

## 1. Introduction

Lung cancer is one of the most common malignant tumors and the leading cause of cancer death. According to the latest global cancer statistics, there were approximately 18.1 million new cancer cases in 2018, including 2.1 million lung cancers, accounting for 11.6%, and 9.56 million new cancer deaths, including 1.76 million lung cancers, accounting for 18.4% [1]. Effective treatments for lung cancer such as surgery, radiotherapy, chemotherapy, targeted therapy, and immunotherapy have greatly improved the prognosis of patients and improved the survival rate of patients, especially the effective rate of targeted therapy for 70–80% [2, 3]. However, lung cancer combined with distant metastasis is still one of the main causes of high mortality in lung cancer [4]. It has been reported that 90% of lung cancer patients die from metastasis, and 70% of patients have lymphatic or distant metastases at the time of initial diagnosis [5].

The invasion and metastasis of lung cancer cells are the main causes of treatment failure and death in patients with lung cancer. The latest research shows that the hypercoagulability is closely related to the development and metastasis of lung cancer [6]. In 1865, Trousseau et al. found that patients with malignant tumors often have a high blood coagulation state [7]. With the advancement of experimental methods and detection methods, a large number of studies have confirmed that platelets participate in multiple steps of tumor metastasis and can promote the growth of primary and metastatic tumors [8]. The study has found that reducing the number of platelets or inhibiting their function can significantly inhibit tumor metastasis [9].

Lung cancer belongs to the category of “xifen,” “feiji,” and “xulao” in Chinese medicine theory. Huayu Wan (HYW) is a prescription of Yiqi Huoxue Jiedu for the core pathogenesis of lung cancer “deficiency”, “stasis,” and “toxin.” A previous clinical study has confirmed that HYW can control tumor growth and reduce the hypercoagulability of patients [10]. In vivo experiments confirmed that HYW inhibits tumor growth and reduces serum P-selectin levels and platelet activation [11–13]. In this study, we intend to explore the mechanism of HYW inhibiting the metastasis of Lewis lung cancer by in vivo experiments in order to provide a theoretical basis for the antitumor metastasis treatment of HYW.

## 2. Materials and Methods

### 2.1. Experimental Animals

A total of 72 C57BL/6 mice (females, 7–8 weeks, 18–20 g, and specific-pathogen-free grade) were purchased from Beijing Vital River Laboratory Animal Technology Co. Ltd. (license no. SCXK (Beijing) 2016-0011) and maintained at the animal facility of the Experimental Animal Research Center of Beijing Chinese Hospital of Traditional Chinese Medicine affiliated to Capital Medical University (license no. SYXK (Beijing) 2018-0006). The mice were housed 6 per cage in an environmentally controlled facility (20–26°C and 40%–70% humidity) with a 12 h light/dark cycle and free access to chow diet and water. All procedures were approved by the Experimental Animal Ethics Committee of Beijing Chinese Hospital of Traditional Chinese Medicine affiliated to Capital Medical University (Ethics no. 2018040204).

### 2.2. Cell Culture

The Lewis lung cancer (LLC) cell line was presented by the Cancer Laboratory of Guang'anmen Hospital, China Academy of Chinese Medical Sciences. LLC cells were cultured in the DMEM-high glucose medium containing 10% FBS, 100 U/mL penicillin, and 100 ug/mL streptomycin at 37°C in a 5% CO_2_ incubator. LLC cells in the logarithmic growth phase were prepared as 1 × 10^7^ cells/mL cell suspension in phosphate-buffered saline (PBS, cell viability was determined by trypan blue staining >98%).

### 2.3. Experimental Drugs and Reagents

HYW is the experience prescription of professor Yu Rencun, a national famous old Chinese medicine doctor. It is also the in-hospital preparation of Beijing Hospital of Traditional Chinese Medicine affiliated to Capital Medical University (Beijing Pharmaceuticals, Z20053296). It is composed of 15 kinds of Chinese herbal medicines, including *Paeoniae Radix Rubra, Curcumae Radix, Ginseng Radix et Rhizoma Rubra*, and *Astragali Radix* [14]. The medicinal materials were provided by the pharmacy of Beijing Chinese Medicine Hospital. Aspirin was purchased from Sigma, USA; TEG, thromboelastography system, kaolin solution, and calcium chloride solution were purchased from Haemonetics, USA; CD62P-FITC, IgG1-FITC, and CD41-PE were purchased from BD Biosciences; and VEGF, CD62P, bFGF, TGF-*β*, and PDGF-AA ELISA kit were purchased from US/Canada RD Corporation.

### 2.4. LLC Metastasis Mice Model

Mice were fixed with mouse clamps, and the tail vein of the mice was sterilized with 75% alcohol and fully exposed to warm light. Cell suspension or PBS (100 uL) was injected horizontally at the tail vein of the mouse. The dry cotton ball was pressed for a while and then returned to the cage until there was no bleeding in the tail vein of the mouse.

### 2.5. Grouping and Administration

On day 2 after inoculation, 72 mice were randomly divided into the control group (*n* = 18), HYW group (*n* = 18), aspirin group (*n* = 18), and normal group by the random number table. Treatment was given for 5 days per week for a total of 16 days. The normal group and the control group were treated by intragastric administration with distilled water. The HYW group was treated by intragastric administration with HYW 23.2 g/kg [13]. In this study, aspirin was used as an antiplatelet positive control, and the dose of aspirin was based on the recommended clinical dosage and some previous studies [15, 16]. Finally, the aspirin group was treated by intragastric administration with aspirin 26 mg/kg.

### 2.6. Weight of Mice Lungs

On the 23rd day after inoculation (pre-experimental results), the mice were anesthetized with 1% pentobarbital sodium by intraperitoneal injection and sacrificed by decapitation. The lung tissue was taken and weighed. And the lung tissues of each group of 3 mice were fixed with Bouin's fixation and photographed.

### 2.7. Thromboelastogram Analysis of Platelet Function

On the 23rd day after inoculation, the mice were anesthetized, blood was collected by the eyeball extraction method, and the mice were sacrificed by decapitation [17]. The anticoagulant after treatment with kaolin and calcium chloride was thoroughly mixed and tested in 4 hours. Thromboelastography was used to detect the whole process of blood clot formation and degradation and quantitatively analyze the platelet function of tumor-bearing mice after different drug interventions.

### 2.8. Flow Cytometry Analysis of Platelet Activation

The whole blood collected from each group of mice was centrifuged at 1500 r/min for 5 minutes at room temperature to separate platelet-rich plasma. Platelet activation was analyzed by flow cytometry after double labeling of platelets by CD62P-FITC and CD41-PE.

### 2.9. ELISA Analysis of Platelet Tumor Metastasis-Associated Factor Expression

The plasma was isolated by centrifuged at 2000 r/min for 20 minutes at room temperature within 4 hours. The expression of CD62P, VEGF, PDGF-AA, TGF-*β*, and bFGF in the serum of each group were detected by ELISA.

### 2.10. Statistical Analysis

Data were collected as mean ± standard deviation (*x* ± *s*). Statistical analysis was performed by SPSS 22.0. The mean of multiple samples was analyzed by variance analysis. *P* values less than 0.05 were considered statistically significant.

## 3. Results

### 3.1. Effect of HYW on Lung Metastasis in LLC Metastasis Mice

**Figure 1:**
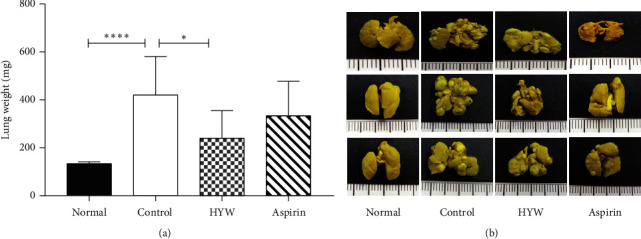
Effect of HYW on lung metastasis in LLC metastasis mice. (a) Lung weight is determined following HYW and aspirin treatment. Columns, mean ± SE; ^*∗∗∗∗*^*P* < 0.0001 and ^*∗*^*P* < 0.05. (b) The lung tissue is immobilized with Bouin's fluid.

On day 23 after inoculation, the lung weight (420 ± 48.54) in the control group was significantly higher than that in the normal group (133.3 ± 2.11) (*P* < 0.05). The lung weight in the HYW group (240 ± 38.37) was lower than that in the control group (*P* < 0.05). The lung weight of the aspirin group was lower than that of the control group (*P* > 0.05), as shown in Figure 1(a). The lung tissue was immobilized with Bouin's fluid as shown in Figure 1(b).

### 3.2. Effect of HYW on Platelet Function in LLC Metastasis Mice

**Figure 2:**
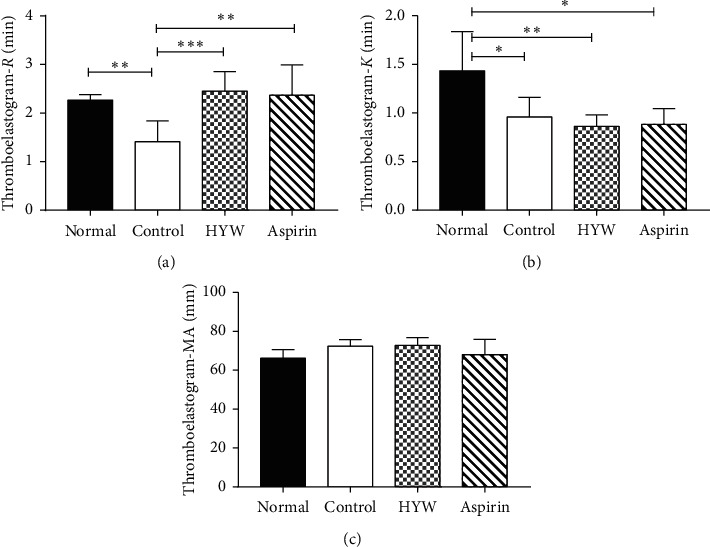
Effect of HYW on platelet function in LLC metastasis mice. (a) *R,* clot reaction time. (b) *K*, time of the speed to reach a certain level of clot strength. (c) Maximum amplitude of maximum strength of the developed clot. Columns, mean ± SE; ^*∗∗∗*^*P* < 0.001, ^*∗∗*^*P* < 0.01, and ^*∗*^*P* < 0.05.

Platelet function was detected by thromboelastography. In the test, we found that the *R* value of the control group was lower than that of the normal group (*P* < 0.05), while the *R* values of the HYW group and the aspirin group were higher than the control group (*P* < 0.05), as shown in Figure 2(a); the *K* value of the normal group was higher than that of the control group, HYW group, and aspirin group (*P* < 0.05), as shown in Figure 2(b); in addition, the MA value of the control group was higher than the normal group, HYW group, and aspirin group, but there was no significant difference between the groups (*P* > 0.05), as shown in Figure 2(c).

### 3.3. Effect of HYW on Platelet Activation in LLC Metastasis Mice

**Figure 3:**
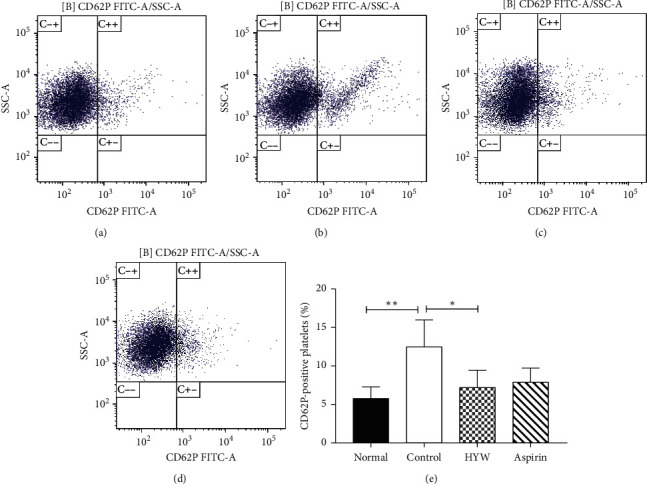
Effect of HYW on platelet activation in LLC metastasis mice. (a) Normal. (b) Control. (c) HYW. (d) Aspirin. (e) Columns, mean ± SE; ^*∗∗*^*P* < 0.01 and ^*∗*^*P* < 0.05.

By flow cytometry, we stained platelet-rich plasma with CD62P and found that the platelet CD62P expression in the control group was significantly higher than that in the normal group (*P* < 0.05). The platelet CD62P expression of the HYW group was lower than the control group (*P* < 0.05). The platelet CD62P expression of the aspirin group was lower than the control group, but there was no significant difference (*P* > 0.05), as shown in Figure 3.

### 3.4. Effect of HYW on the Platelet-Associated Tumor Metastasis Factor in LLC Metastasis Mice

**Figure 4:**
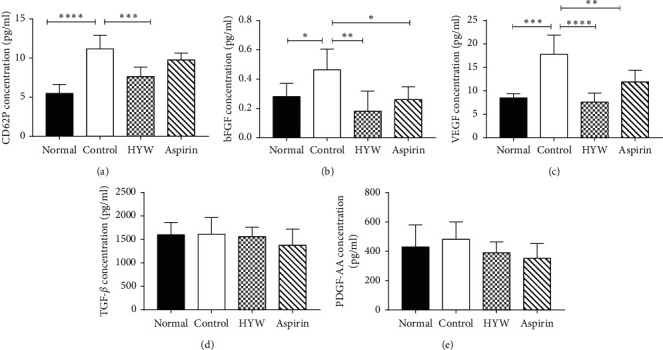
Effect of HYW on the platelet-associated tumor metastasis factor in LLC metastasis mice. (a) CD62P, (b) bFGF, (c) VEGF, (d) TGF-*β*, and (e) PDGF-AA. Columns, mean ± SE; ^*∗∗∗∗*^*P* < 0.0001, ^*∗∗∗*^*P* < 0.001, ^*∗∗*^*P* < 0.01, and ^*∗*^*P* < 0.05.

As shown in Figures 4(a)−4(e), the expression of CD62P in the control group was higher than the normal group (*P* < 0.05), while the expression of CD62P in the HYW group was lower than the control group (*P* < 0.05). The expression of bFGF and VEGF in the control group was higher than that in the normal group (*P* < 0.05), while HYW and aspirin could inhibit the expression of bFGF and VEGF in different degrees (*P* < 0.05). However, the expression of TGF-*β* and PDGF-AA was not significantly different between the groups in this study (*P* > 0.05).

## 4. Discussion

Lung cancer is the malignant tumor with the highest mortality rate and the third highest incidence rate in the world. It is also the malignant tumor with the highest morbidity and mortality in China [18]. Distant metastasis is the main cause of death in patients with lung cancer. The occurrence and development of lung cancer are closely related to the hypercoagulable state.

Platelets are small, nonnuclear blood cell fragments that are lysed and detached from bone marrow megakaryocytes. They are present in blood circulation and play an important role in the formation of hypercoagulable blood and tumor metastasis.

Platelets participate in multiple steps of tumor metastasis and promote the growth of primary and metastatic tumors. The research reveals that its main mechanisms include the following: (1) tumor cell-induced platelet aggregation (TCIPA) provides the possibility for tumor cells to survive and metastasize in the blood [19]; (2) platelets form tumor complexes with tumor cells, protect tumor cells from blood flow damage, and escape the body's immune system; (3) adhesion molecules integrin and P-selectin on the platelet membrane not only promote homologous adhesion of platelets to blood vessel walls but also promote heterotypic adhesion of tumor cells to blood vessel walls [20, 21]; and (4) activated platelets release a large number of angiogenic regulatory substances such as vascular endothelial growth factor (VEGF), platelet-derived growth factor (PDGF), and transforming growth factor (TGF-*β*), which are involved in tumor growth and metastasis [22].

In this study, the lung metastasis model was constructed by injecting LLC cells into the tail vein of C57BL/6 mice, and the transfer rate was 100%. In addition, in the caudal-vein lung metastasis model we constructed, we found that late lung metastasis was relatively serious. Many tumors have fused and even metastasized to the pleura and bronchus. Considering that the number of metastases could not objectively reflect the actual metastasis and referring to previous research studies [23], we finally chose lung weight to reflect lung metastasis. The lung weight decreased compared with the model group after treatment with HYW, suggesting that HYW can effectively inhibit the metastasis of LLC cells. As one of the most commonly used nonsteroidal anti-inflammatory drugs, aspirin has antiplatelet effects [24], promotes fibrinolysis [25], and reduces the risks of cancer [26]. However, there was no statistically significant difference in the inhibition of LLC cell metastasis by aspirin. The thromboelastogram is a dynamic observation of the blood coagulation process. From the beginning of coagulation, the formation of blood clots to the process of fibrinolysis, a comprehensive assessment of coagulation factors, fibrinogen, and platelet function can be performed [27]. The *R* value is the time of the coagulation reaction, that is, the time required for the blood clot to start to form from the beginning of the test sample. The prolongation of the *R* value may be a deficiency of the coagulation factor, anticoagulation, or severe fibrinogenemia. Conversely, shortening of the *R* value indicates that blood is hypercoagulable [28]. The results of this experiment showed that the *R* value of the control group was significantly lower than the normal group, and the *R* values of the HYW and aspirin groups were higher than the control group, suggesting that HYW and aspirin can improve the hypercoagulable state of blood. CD62P is mainly present in platelet *α*-particles and is an important marker for platelet activation [29]. A prospective cohort study confirmed that platelet activation was significantly associated with the lung cancer risk by the multivariate Cox regression model and that CD62P is an independent risk factor for lung cancer [30]. Flow cytometric analysis indicated that the expression of CD62P was increased in the control group compared with the normal group, and the expression of CD62P in HYW and aspirin groups was lower than the control group, suggesting that HYW and aspirin intervention can inhibit platelet activation to varying degrees.

In addition, we examined the expression of platelet-associated tumor metastasis factors by ELISA. Vascular endothelial growth factor (VEGF) is a cytokine that promotes vascular endothelial growth and plays an important role in angiogenesis, promoting endothelial cell proliferation, migration, and invasion [31]. It promotes tumor development through autocrine or paracrine mechanisms [32]. Fibroblast growth factor-2 (bFGF) has a wide range of biological activities, including promoting wound healing, promoting neovascularization, and repairing damaged blood vessels, and is highly expressed in many tumor tissues, and its expression may be related to the formation of tumor blood vessels [33]. The results of ELISA showed that the expression of bFGF and VEGF in the control group was higher than that in the normal group (*P* < 0.05), while HYW and aspirin could inhibit the expression of bFGF and VEGF in different degrees (*P* < 0.05). At the same time, the CD62 results of the ELISA assay are consistent with the results of the flow cytometry.

## 5. Conclusions

In summary, HYW could inhibit the metastasis of LLC cells, and its mechanism may be related to the improvement of blood hypercoagulability, inhibition of platelet activation, and downregulation of VEGF and bFGF expressions.

## Data Availability

The data that support the findings of this study are available from the corresponding author upon reasonable request.
